# Incidental Renal Langerhans Cell Histiocytosis Within Clear Cell Renal Carcinoma: A Case Report and Literature Review

**DOI:** 10.1155/criu/4441127

**Published:** 2025-11-10

**Authors:** Charles-Antoine Garneau, Cathie Ouellet, Sophie Morin, Isabelle Harvey, Bruno Turcotte

**Affiliations:** ^1^Urology Division, Department of Surgery, CHU de Québec-Université Laval, Quebec, Quebec, Canada; ^2^Faculty of Medicine, Université́ Laval, Quebec, Quebec, Canada; ^3^Department of Pathology, CHU de Québec-Université Laval, Quebec, Quebec, Canada

## Abstract

Langerhans cell histiocytosis (LCH) is a disease characterized by the excessive proliferation and abnormal differentiation of immune cells, including monocytes, T cells, and dendritic cells. The most affected organs are the bones, skin, lungs, liver, and spleen, while renal involvement is rare. LCH primarily affects children and is seldom diagnosed in adults. In this case report, we describe a 64-year-old man patient with no prior urological history nor history of LCH and presenting with both localized renal LCH and clear cell renal carcinoma (ccRCC). Renal cancer was first discovered fortuitously on an abdominal computed tomography (CT) scan while the patient presented symptoms of a complicated urinary tract infection. Following radical nephrectomy, LCH foci was detected as incidental histological finding within the ccRCC pathological analysis. Immunohistochemical staining confirmed the positivity of S-100 and CD1a markers and PCR analysis identified the BRAF^V600E^ mutation. Based on these findings, a diagnosis of ccRCC associated with LCH was established.

## 1. Introduction

Langerhans cell histiocytosis (LCH) is a rare disorder characterized by the abnormal proliferation of immature Langerhans cells, which are a type of dendritic cells. This pathology can be localized or systemic and can manifest in multiple organs [1]. Bones are most affected, although involvement of the skin, endocrine glands, lungs, liver, spleen, and central nervous system has also been reported. Renal involvement, however, remains exceedingly rare and has been generally documented in patients with multisystem disease. While LCH is predominantly observed in children, adults can also be affected but usually presents disseminated disease [2].

On the other hand, clear cell renal cell carcinoma (ccRCC) is the most common type of kidney cancer and makes up about 80% of all renal cell carcinoma cases [3]. This cancer typically manifests as a solitary tumor differentiating toward a proximal tubule phenotype. Certain risk factors such as obesity, hypertension, and tobacco use are involved in pathogenesis.

The association between LCH and various malignant tumors has been documented in several case reports and studies [4–6]. However, only a handful of cases have described renal infiltration by LCH and the coexistence of LCH with ccRCC is even more uncommon [7, 8]. The relationship between these two conditions remains poorly understood in literature, and several hypotheses have been proposed to explain this association. This case report presents an instance of LCH coexisting with ccRCC in an adult man case.

## 2. Case Presentation

A 64-year-old man with no previous urological history presented to the emergency room with acute left testicular pain. In addition to the testicular pain, the patient reported gross hematuria, pollakiuria, dysuria, and fever, all of which had been present for 8 h prior to seeking medical attention. The patient's medical history included active smoking with 23 pack-years and a prior inguinal hernia surgery 25 years ago. He had no significant family history.

Upon examination, the patient was hemodynamically stable but presented with a fever of 39.4°C. Laboratory tests, including a complete blood count and metabolic panel, revealed an elevated white blood cell count, but kidney function was normal, with a creatinine level of 88 *μ*mol/L. Urinalysis showed both white and red blood cells, nitrites, and bacteria. Physical examination revealed a nontender and painless abdomen and a negative renal punch. The scrotum appeared normal, but the left testis was inflamed and painful. Rectal examination revealed exquisite tenderness upon palpation of an indurated, enlarged, symmetrical prostate.

A complicated urinary tract infection, with prostatitis and orchiepididymitis, was suspected. To rule out an infected kidney stone, a noncontrast abdominal computed tomography scan was performed. The CT scan revealed a deforming, partially exophytic 3.4 × 4.8 × 4.5 cm right renal mass, consistent with a diagnosis of a renal malignancy. Additionally, a nonobstructive, coralliform kidney stone measuring 11 mm was identified in the left lower pole. The urine culture, obtained a few days later, confirmed an *Escherichia coli* infection. The patient was treated with a 6-week course of levofloxacin and scheduled for follow-up.

To further assess the renal mass, an abdominal ultrasound was performed 2 weeks later. The ultrasound revealed a hyperechoic, vascularized solid mass at the posterior edge of the right kidney. A multiphased abdominal CT scan conducted 1 week later confirmed the presence of a heterogeneous, enhancing 5.3 × 3.5 × 5.8 cm mass located in the midportion of the right kidney, consistent with renal cell carcinoma. The renal artery and vein were patent, and there was no evidence of hydronephrosis in either kidney. No lymphadenopathy or metastases were observed. Further evaluation included a thoracic CT scan and bone scan. The bone scan was negative for osseous lesions, but a small nonspecific nodular lesion was detected on the thoracic CT scan, requiring radiologic follow-up within 6 months.

The patient underwent a right laparoscopic radical nephrectomy, 2 months after the suspected renal malignancy was made based on radiologic studies. Preoperatory laboratory investigations were unremarkable. Indeed, neutrophils had normalized and there were no eosinophils or monocytes. The surgery was uneventful, and the patient was discharged 2 days after the surgery without any complications.

Following nephrectomy, pathological analyses of the kidney were performed in our tertiary academic center. Gross examination of the specimen showed a yellow, well demarcated mass of 5 × 4.2 × 2.6 cm localized in the middle tier/lower pole of the kidney, without necrosis or hemorrhage. Histology of the pathological sample demonstrated Grade 2 clear cells renal carcinoma. Resection margins were clear of carcinoma. There was no lymphovascular invasion. The final pathologic stage (based on the AJCC 8e edition) was pT1bNx.

**Figure 1:**
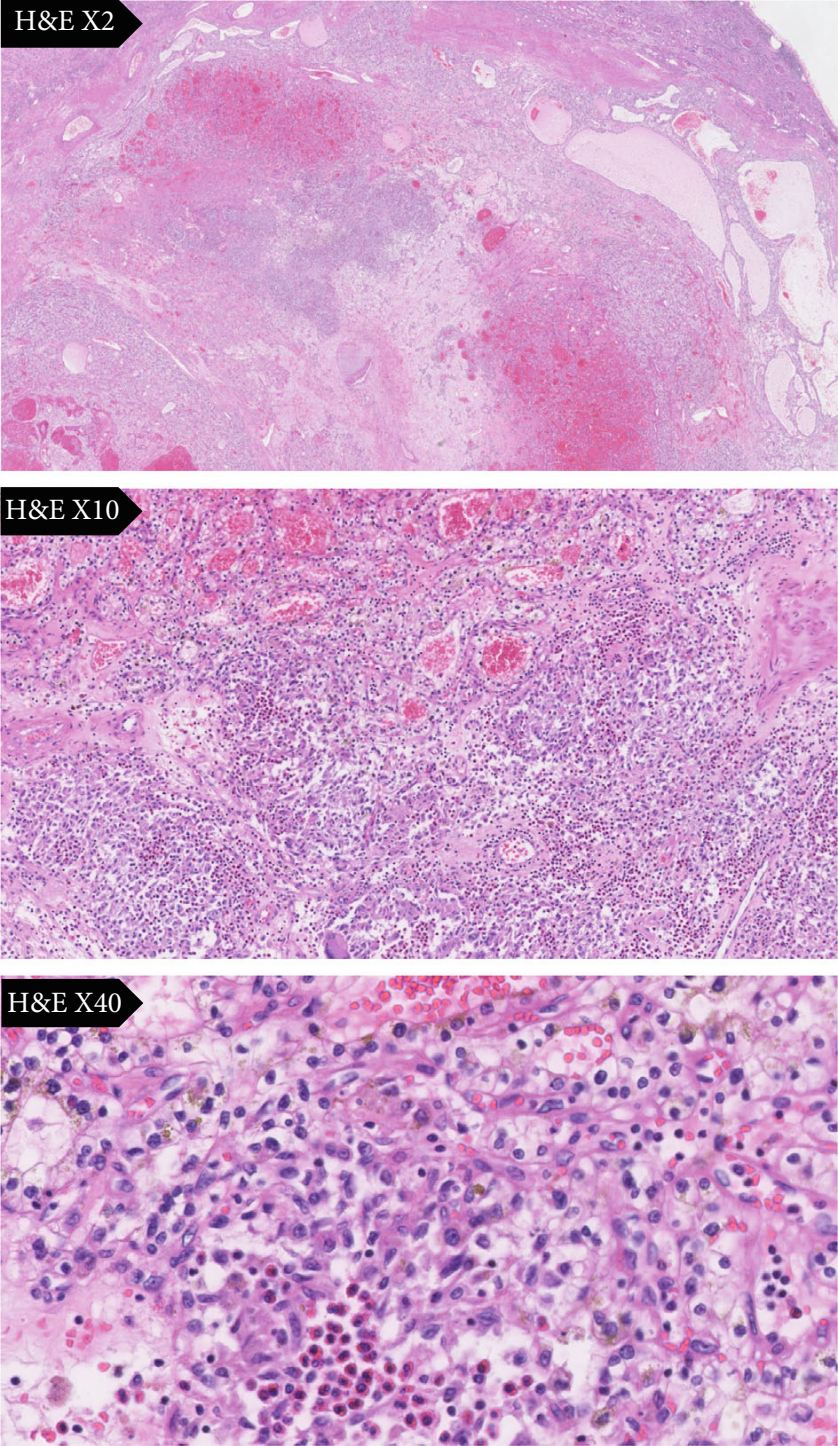
Photomicrographs showing incidental histological findings of Langerhans cells and eosinophils admixed within the resected renal specimen surrounded by the ccRCC. H&E.

**Figure 2:**
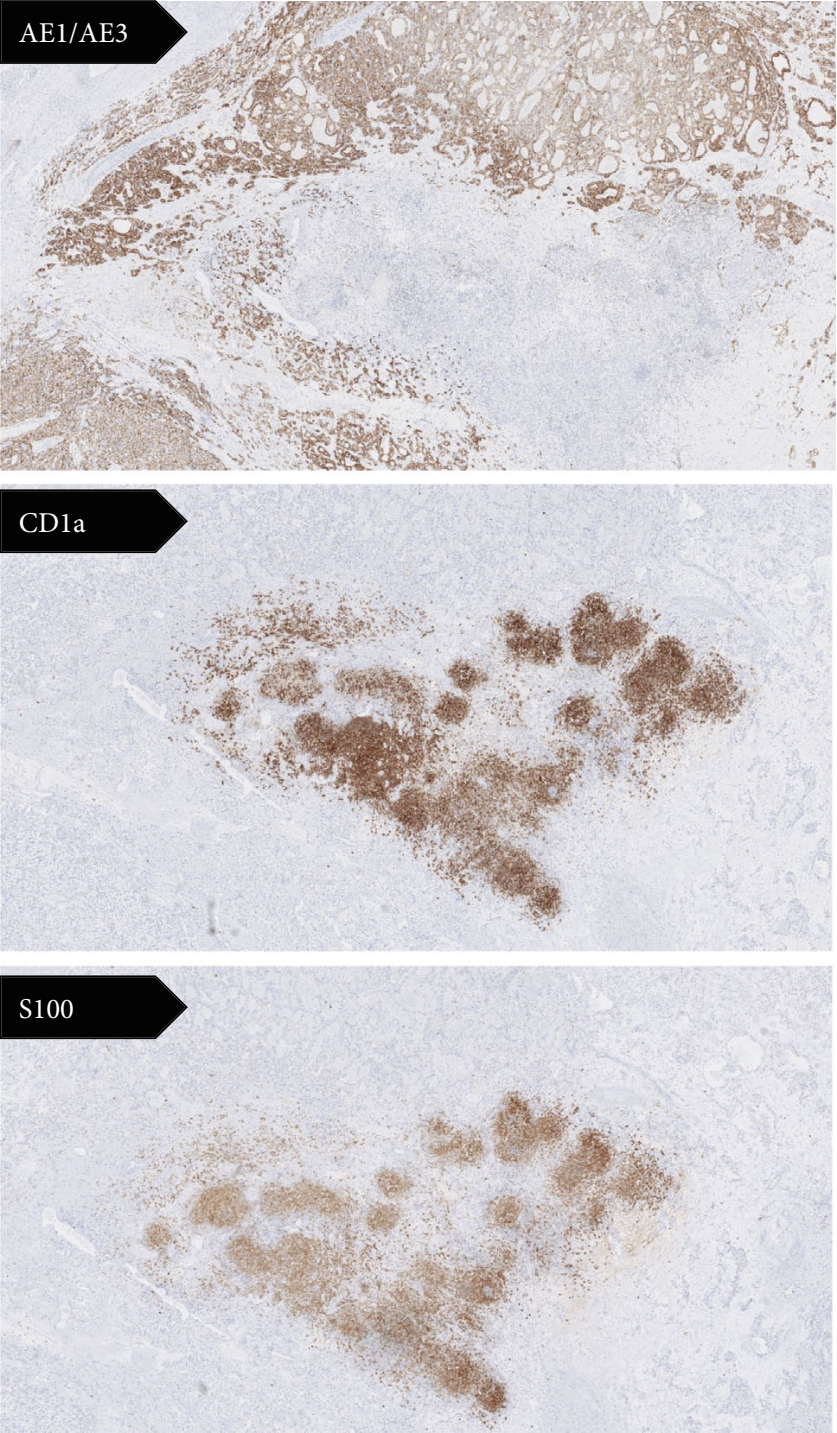
Photomicrographs showing immunostaining positivity for S-100 and CD1a and negativity for AE1/AE3 suggesting Langerhans cell histiocytosis. X2.

One LCH focus, of approximately 5 mL, has been discovered as an incidental histological finding within the resected renal specimen surround by the ccRCC (Figure 1). Immunohistochemical stains revealed positivite for S-100, CD1a, vimentin, and CD68 and negative for AE1/AE3 (Figure 2). Moreover, the detection of BRAF mutations by the allele-specific PCR technique showed a BRAF^V600E^ mutation. Upon thorough examination, no evidence of LCH was observed in the normal kidney tissue or the surrounding perinephric adipose tissue. The LCH lesion was confined exclusively to the zone of the renal cell carcinoma. Thereby, the final diagnosis was ccRCC with secondary involvement by LCH.

A hematology consultation was carried out 6 months after the patient's surgery to investigate the presence of other possible systemic symptoms. On further screening workup and follow-up examination, no other LCH manifestations were detected. Clinically, the patient remained asymptomatic afterwards and was kept under surveillance without any further treatment. More than 18 months after the diagnosis, the patient shows no signs of LCH elsewhere.

## 3. Discussion

LCH is an extremely rare and complex disease occurring in approximately 0.07 per million cases annually [9]. Adult LCH typically manifests after patients reach their 40s, with approximately two-thirds of patients presenting with multisystem disease at the time of initial diagnosis [10]. The disease, characterized by the predominant activation of innate immune cells, leads to the infiltration of large numbers of Langerhans cells in the affected organs [11].

Classical immunohistochemical characteristics include clonal proliferation of mature dendritic cells with the expression of CD1a, S100 protein, and CD207 (langerin) which is associated with the presence of distinctive cytoplasmic Birbeck granules [12]. Furthermore, Geissmann et al. were the first to propose that a blockage in the maturation pathway of LCH cells could be caused by interactions between langerin-positive cells and inflammatory cells, resulting in immature LCH cell function [2]. Various studies have highlighted that a cytokine storm plays a central role in the development of LCH. Specifically, interactions between Langerhans cells and other immune cells, such as T cells, macrophages, and neutrophils, amplify cytokine secretion, leading to the recruitment and proliferation of LCH-associated cells [13, 14].

While LCH was previously considered primarily an inflammatory disorder, recent research has led to its reclassification as a myeloid neoplasm with inflammatory features [15]. Under normal physiological conditions, Langerhans cells play a crucial role in facilitating communication between various immune cells. However, genetic mutations, such as the BRAF^V600E^ mutation, as well as mutations that aberrantly activate the MAPK/ERK signaling pathway, can lead to the hyperproliferation and abnormal survival of LCH cells and the activation of other immune cells, including T cells, eosinophils, and neutrophils. This results in the invasion of surrounding tissues [16]. The BRAF ^V600E^ mutation is a well-established driver mutation in LCH, and the most common genetic alteration present in approximately 50%–60% of cases [17]. This mutation is more often identified in patients with multisystem disease and those with more severe clinical presentation [18].

The association between LCH and malignant tumors is uncommon, with most reported cases involving lymphomas and leukemias [4]. The pathogenesis of LCH in the context of malignancy remains poorly understood. The occurrence of LCH in association with other malignancies exhibits diverse temporal and spatial patterns. In some cases, LCH may develop as a secondary response to chemotherapy or radiotherapy, or in another organ as a reactive process to a malignancy. We were able to identify a dozen documented cases of LCH coexisting with RCC. A recent case series described 10 cases of patients with LCH occurring within ccRCC, similar to the present case [7]. The simultaneous presence of both tumors has been described too often to be an unrelated coincidence.

The underlying mechanisms driving the association between LCH, and other neoplasms remain largely enigmatic, and several hypotheses have been proposed. Most authors speculate that the tumor microenvironment may produce a unique profile of cytokines, chemokines, and growth factors that could promote the recruitment, proliferation, and survival of Langerhans cells or their precursors. Indeed, RCC are known to produce diverse cytokines such as IL-6, TNF-*α*, and VEGF, which could stimulate the growth and differentiation of LCH cells. Overexpress growth factors like TGF-*β* and PDGF in RCC could support the proliferation of LCH cells [19]. ccRCC is also known as an immunogenic tumor characterized by a sophisticated ability to induce adaptive immune responses by the recruitment of T cells and tumor-associated macrophages [20]. The immunosuppressive environment created by the RCC might alter immune surveillance, allowing for the unchecked growth of LCH lesions. Finally, the presence of tumor antigens might trigger an aberrant proliferation of Langerhans cells as part of a dysregulated immune reaction [15].

Common underlying environmental risk factors might increase susceptibility to both conditions. Indeed, the potential contribution of extrinsic factors, particularly cigarette smoking, cannot be overlooked in the development of both RCC and LCH. Smoking is a well-established potent and dose-dependent risk factor of RCC. The relative risk is estimated at more than two times for those with more than 20 pack-years [21]. Cigarette smoke contains numerous carcinogens, including polycyclic aromatic hydrocarbons and beta-naphthylamine. When metabolized, these compounds promote inflammation and induce DNA damage, contributing to carcinogenesis [22]. Furthermore, smokers or individuals with a history of tobacco use are more likely to develop aggressive forms of RCC, and they often exhibit a poorer response to treatment [23]. The strong association between smoking and LCH is also well established, particularly with pulmonary Langerhans cell histiocytosis (PLCH). The exact mechanisms linking smoking to PLCH development are still not fully understood, but over 95% of the PCLH cases occur in smokers [10]. While previously thought to be a reactive condition associated with smoking, it is now considered a trigger or promoter rather than the sole cause of the disease. Current evidence suggests that cigarette smoke triggers a complex interplay of inflammatory and neoplastic processes and leads to an increase in Langerhans cells, particularly in the lungs. Tobacco-derived antigens, such as bombesin-like peptides and tobacco glycoprotein, activate macrophages to secrete inflammatory cytokines like TNF-alpha, GM-CSF, and TGF-*β*, as well as the recruitment and activation of Langerhans cells [24, 25]. Additionally, the accumulation of inflammatory cells, combined with dysfunctional apoptosis and accelerated cell proliferation, has been linked to LCH [26].

Shared genetic predispositions could also explain this coexistence. Recent studies have shown that in RCC, the MAPK signaling pathway enhances the expression of Matrix Metalloproteinase 2 (MMP-2) [27]. This suggests that the MAPK pathway could be involved in the development of both LCH and RCC, particularly in this patient who was positive for the BRAF^V600E^ mutation. PCR testing revealed the BRAF^V600E^ mutation in the present patient and in most but not all reported cases, leading some authors to conclude to a reactive-inflammatory process [8, 28]. However, the scientific community seems to agree that the presence of BRAF^V600E^ mutations provides compelling evidence of genuine neoplastic process, rather than reactive process. Agaimy et al. have reported that the frequency of BRAF^V600E^ mutations in LCH seems to vary based on the method used for analysis, which could explain the inconsistent detection of the mutation [7]. That being said, some data suggest a common mechanism of activation of the signal transduction pathway, regardless of BRAF mutation status [29]. According to our literature review, approximately 70% of RCCs patients tested for the BRAF^V600E^ mutations were positive. Additionally, the positivity of Cyclin D1, a downstream marker of MAPK, suggests the potential involvement of a neoplastic origin [30]. These genetic alterations result in constitutive activation of the MAPK pathway, leading to abnormal proliferation of LCH cells. The identification of these driver mutations has not only enhanced our understanding of LCH pathogenesis but also opened new avenues for targeted therapies, such as BRAF and MEK inhibitors.

In the present case, the patient was an active smoker with more than 20 pack-year similarly to other reported cases. Cigarette smoking could certainly have served as a potential trigger. However, the presence of the BRAF V600E mutation and cyclin D1 expression indicates that it may be more of a neoplasic process than reactive-inflammatory. It is important to emphasize that in the majority of reported cases, LCH seems to arise within the RCC, and the remaining nonneoplastic renal parenchyma was devoid of Langerhans cells. Furthermore, all cases were low-grade ccRCC with variable cystic changes [31]. Our patient was in the same age range as other described cases. However, Tran and Mekhail noted that this phenomenon occurred more in the left kidney, which is not the case here [32]. As in most reported cases, follow-up examinations revealed no evidence of progression or widespread dissemination of the LCH after the resection of the RCC. This implies a potential dependence of the LCH on the microenvironment provided by the RCC and a more indolent nature of the disease. However, given the unpredictable nature of LCH, long-term surveillance remains essential to detect any late recurrence or progression.

## 4. Conclusion

Herein, we presented a rare case of occult LCH associated with RCC, which appears to be a neoplastic process rather than a reactive histiocytic proliferation. Indeed, the identification of the BRAF mutation in this case aligns with the current understanding of LCH as a clonal myeloid neoplasm driven by activating mutations in the MAPK pathway. This case report suggests that this phenomenon may be more prevalent than currently reported in the literature and may be unseen by pathologists if the lesion is not well sampled. Further research, including comprehensive molecular profiling of concurrent LCH and malignant lesions, analysis of the tumor microenvironment, and longitudinal studies to determine the temporal relationship between these conditions, are essential to better understand the implications of this coexistence. An appropriate follow-up is recommended no matter the reactive, coincidental, or neoplastic etiology.

## Data Availability

All data are contained within the manuscript.
